# Molecular Dynamic Simulation of Space and Earth-Grown Crystal Structures of Thermostable T1 Lipase *Geobacillus* *zalihae* Revealed a Better Structure

**DOI:** 10.3390/molecules22101574

**Published:** 2017-09-25

**Authors:** Siti Nor Hasmah Ishak, Sayangku Nor Ariati Mohamad Aris, Khairul Bariyyah Abd Halim, Mohd Shukuri Mohamad Ali, Thean Chor Leow, Nor Hafizah Ahmad Kamarudin, Malihe Masomian, Raja Noor Zaliha Raja Abd Rahman

**Affiliations:** 1 Enzyme and Microbial Technology Research Centre, Faculty of Biotechnology and Biomolecular Sciences, Universiti Putra Malaysia, 43400 UPM Serdang, Selangor, Malaysia; hasz_usm@yahoo.com (S.N.H.I.); snariati@gmail.com (S.N.A.M.A.); mshukuri@upm.edu.my (M.S.M.A.); adamleow@upm.edu.my (T.C.L.); hafizah_kamar@upm.edu.my (N.H.A.K.); malihe.m@upm.edu.my (M.M.); 2 Department of Cell and Molecular Biology, Faculty of Biotechnology and Biomolecular Sciences, Universiti Putra Malaysia, 43400 UPM Serdang, Selangor, Malaysia; 3 Department of Biotechnology, Kuliyyah of Science, International Islamic University Malaysia, Bandar Indera Mahkota, 25200 Kuantan, Pahang, Malaysia; kbariyyah@iium.edu.my; 4 Department of Biochemistry, Faculty of Biotechnology and Biomolecular Sciences, Universiti Putra Malaysia, 43400 UPM Serdang, Selangor, Malaysia; 5 Institute of Bioscience, Universiti Putra Malaysia, 43400 UPM Serdang, Selangor, Malaysia; 6 Department of Microbiology, Faculty of Biotechnology and Biomolecular Sciences, Universiti Putra Malaysia, 43400 UPM Serdang, Selangor, Malaysia; 7 Laboratory of Halal Science Research, Halal Products Research Institute, Universiti Putra Malaysia, 43400 UPM Serdang, Selangor, Malaysia

**Keywords:** T1 lipase, *Geobacillus zalihae*, microgravity, molecular dynamic simulation, hydrogen bond, ion interaction

## Abstract

Less sedimentation and convection in a microgravity environment has become a well-suited condition for growing high quality protein crystals. Thermostable T1 lipase derived from bacterium *Geobacillus* *zalihae* has been crystallized using the counter diffusion method under space and earth conditions. Preliminary study using YASARA molecular modeling structure program for both structures showed differences in number of hydrogen bond, ionic interaction, and conformation. The space-grown crystal structure contains more hydrogen bonds as compared with the earth-grown crystal structure. A molecular dynamics simulation study was used to provide insight on the fluctuations and conformational changes of both T1 lipase structures. The analysis of root mean square deviation (RMSD), radius of gyration, and root mean square fluctuation (RMSF) showed that space-grown structure is more stable than the earth-grown structure. Space-structure also showed more hydrogen bonds and ion interactions compared to the earth-grown structure. Further analysis also revealed that the space-grown structure has long-lived interactions, hence it is considered as the more stable structure. This study provides the conformational dynamics of T1 lipase crystal structure grown in space and earth condition.

## 1. Introduction

Protein crystallization is vital to the illumination of the three-dimensional structures of enzymes. Understanding these structures in turn allows for better understanding of the mechanisms and structural functions of proteins. The primary objective of the crystallization process is the production of well-ordered high-quality monocrystals which are large enough to provide a diffraction pattern during X-ray diffraction analysis. Over the past decade, crystallization of proteins and biological components such as nucleic acids, nucleosomes, viruses, ribosomal subunits, or nucleo-protein complexes under microgravity environment (~10^−3^–10^−6^× *g*) has attracted special attention. The reduction of buoyancy-driven convection and lack of sedimentation under microgravity condition are the ideal for macromolecular crystal growth. These factors provide better conditions for the formation of high quality protein crystals with better internal orders [1]. Crystallization of macromolecules under microgravity conditions has been shown to improve the size, perfection, morphology, and internal order of protein crystals [2]. Such microgravity-grown crystals can be diffracted to a high resolution. They have a lower mosaicity, which defines their quality. The quality of the crystal is important, as it comprises the details of the electron density map [1].

Molecular dynamics (MD) simulation has become the foremost tools to study the structure-function relationship and molecular arrangement of proteins. MD simulation has been extensively used to draw biological and physiological conclusion of the structure-function relationships of membrane transport protein using the homology models, X-ray, and NMR structural data under various resolutions [3] and also to refine the protein structure models [4]. MD simulation provides information on the fluctuations and conformational changes of macromolecules. This method is now also used to investigate the structure, dynamic, and thermodynamic of biological macromolecules. This method is an alternative way to explore the behavior of protein under certain conditions which are impossible to establish in the laboratory. Hence, MD simulation makes it possible to study the conformational assembly of proteins and achieve insight into molecular mechanisms.

A gene encoding for thermoalkalophilic lipase from *Geobacillus zalihae* was previously cloned and expressed in an *Escherichia coli* system [5]. The crystal structure of T1 lipase (2DSN) was obtained using the hanging drop vapor diffusion method at a resolution of 1.5 Å [6]. T1 lipase was successfully crystallized using the counter diffusion method under microgravity environment in collaboration with JAXA under the JAXA-UPM Protein Crystal Growth (PCG) #2 Flight Program [7]. The production of good quality crystals under space condition and their characteristics have been previously discussed [7]. However, there has been limited discussion on discrepancies among the structural features of space and earth-grown crystal structures. Here we investigate the detailed structural architecture of the space and earth-grown crystal structure of T1 lipase using an MD simulation approach. For the first time, MD simulation has been performed to allow detailed comparison of the structures and confirmed the differences in space- and earth-grown T1 lipase. The intermolecular interactions and conformational structure that contribute to the stability of the protein are compared and discussed.

## 2. Results and Discussions

### 2.1. Preliminary in Silico Analyses of T1 Lipase Crystal Structures of Space- and Earth-Grown

Previously, T1 lipase crystals obtained from space and earth were solved to a resolution of 1.1 Å and 1.3 Å, respectively [7]. Both T1 lipase structures consist of two molecules per asymmetric unit and are referred to as chain A and chain B. Each molecule consists of 387 amino acids, starting with Ser2. The catalytic triad of T1 lipases consists of residues Ser113, Asp317, and His358. The crystal structure of earth-grown T1 lipase showed a root mean square deviation (RMSD) of 0.2185 Å when superimposed with the space-grown crystal structure (Figure S1). Both structures were highly similar, with only minor differences.

**Figure 1:**
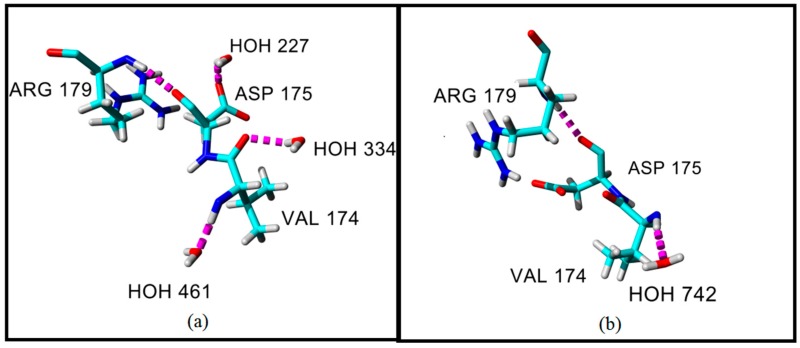
The conformation of residues Asp175, Arg179, and Val174 in T1 lipase crystal structures: (**a**) earth-grown T1 lipase crystal structure; (**b**) Space-grown T1 lipase crystal structure.

Variations were determined in the conformation of residue Asp175 (Figure S2). The region of Asp175 in an earth-grown crystal structure faces towards the core of the protein structure and the distance of the peptide bond between the residues of Val174 and Asp175 in the earth-grown structure is 3.57 Å as compared with the same residues in a space-grown structure (3.78 Å). More detailed inspection of the structure has shown that Asp175 in the earth-grown structure formed a hydrogen bond only with Arg179 and one water molecule (Figure 1a). While, the space-grown crystal structure formed an interaction with Arg179. The residue of Val174 next to Asp175 in the earth-grown structure was found to interact with two water molecules while in the space-grown crystal structure, only one interaction was observed with one water molecule (Figure 1b).

Early experiments showed that crystals grown in space conditions were uniform, high quality, and larger than crystals grown on earth. There are four criteria involved in comparing crystal grown in space with equivalent crystals grown on earth: subjective visual quality; maximum size and size distribution; morphology; and X-ray diffraction quality. The microgravity environment has been found to be an excellent environment for the formation of a high quality crystal with explicit information and translation of its protein structure. In agreement, crystal structure of sperm whale myoglobin triple mutant Mb-YQR derived from space condition provided high quality diffraction data which contributes to a very accurate and precise model structure. Miele et al. [8] revealed that space-grown crystals diffract to better resolution, allowing substantially more precise X-ray diffraction data than earth-grown crystals. Likewise, more features are visible in the electron density map of aminoacyl-tRNA synthethase crystal structure correlative to the polypeptide backbone and its side chain. Therefore, well defined amino acids and a higher order of bound water molecules are visible in the space-grown crystal [9]. Particularly, comparisons of structural data from space- and earth-grown crystals structures of acidic phospholipase A2 concluded that the microgravity does not modify the conformation of the polypeptide chains of proteins. However, it showed some improvement in the bound water structure at the hydration layer. This may be an important factor in the quality of protein crystal grown under space conditions [10]. Microgravity environments are excellent environs for the formation of high quality crystal with high resolution and lower mosaicity. The results obtained from previous research are encouraging, however the question remains whether space-grown crystals can be useful for the determination of three-dimensional structures remained open.

### 2.2. Molecular Dynamic Study of Space- and Earth-Grown Structures

Space crystallization also provides better information in structural architecture and conformational structure as shown in this study. The pilot analysis of protein structures derived from space- and earth-grown crystal structures showed some differences. Hence, a molecular dynamic simulation study was employed to investigate and validate the discrepancy of the structures. In this paper, the differences of the intermolecular interactions and conformation of both three-dimensional structures have been studied using MD simulation approach. This computer simulation method calculates the time dependent behavior of the molecular system. Previously, Groot et al. [11] showed that MD simulation of T4 lysozyme provided a reliable prediction of its functional dynamics. Law et al. [3] concluded that MD is capable of differentiating both the quality and stability of two similar models. MD simulation is used to obtain detailed information of the effect of high pressure on protein and was applied to disclose similarities and differences between deep- and shallow-sea protein models at different temperatures and pressures [12,13]. In this study, the comparison of both structures showed the improvement of structure stability and structural architecture in the space-grown structure. The improvement of protein crystals quality from space condition may be related to the solvent content of the protein crystal [10]. Since fluid convection-driven motion around the crystalizing protein depends on the level of gravity, the total number of the bound water molecules may concurrently vary.

**Figure 2:**
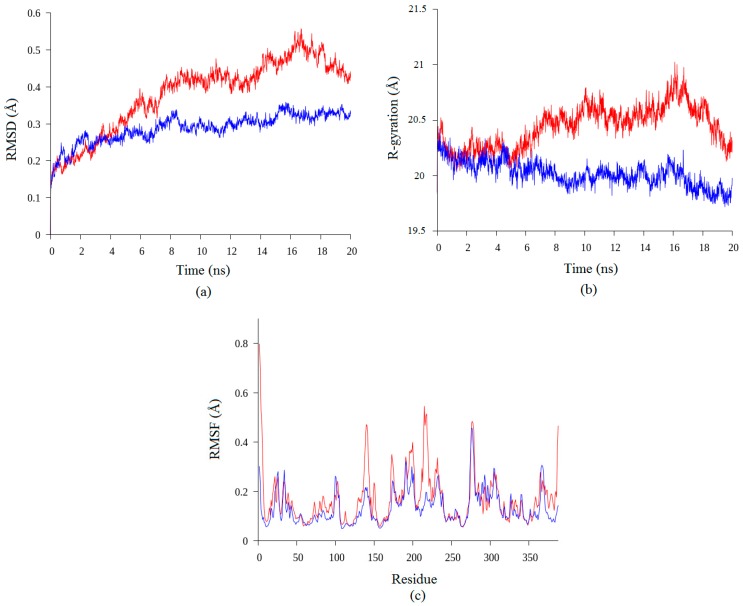
Analysis of molecular dynamic simulation of earth-grown (red) and space-grown (blue) crystal structures in water at 70 °C: (**a**) The Root Mean Square Deviation (RMSD) of earth-grown and space-grown T1 lipase crystal structures; (**b**) Radius of gyration of earth-grown and space-grown T1 lipase crystal structures; (**c**) Root Mean Square Fluctuation (RMSF) of earth-grown and space-grown T1 lipase crystal structure.

According to Pikkemaat et al. [14], the stability of protein can be analyzed using MD study by evaluating the RMSD of the protein structure and the stability of secondary structure elements. Based on the simulation results presented in Figure 2, both of the structures showed some increment in RMSD values during the simulation. High values of RMSD showed the structural changes during the simulation which can be used to identify quality of the structure [3]. The RMSD value of the earth-grown structure increased from 0.15 Å until 0.4 Å for the first 6 ns of simulation. The RMSD of the structure was stable at 0.4 Å until 14 ns of simulation and it increased again until 0.5 Å. At the end of the simulation, the RMSD value of the earth-grown structure decreased to 0.4 Å. Conversely, the RMSD value of the space-grown structure fluctuated during the first 3 ns of simulation, but was then stable with an average of 0.3 Å until the end of simulation. Our results showed that the earth-grown structure endured minor conformational changes during the MD simulations as shown in Figure 2a. The increasing value of RMSD in earth-grown is due to enhanced motions between the atoms. The space-grown structures have thus been shown to have better conformational relative stablitiy than earth-grown structures. RMSD is the most commonly used quantitative measure of the similarity between two superimposed atomic coordinates [15].

In addition to the study, the analyses of the radius of gyration and the Root Mean Square Fluctuation (RMSF) have been conducted. In agreement with the RMSD results, the earth-grown structure also showed increasing value of radius of gyration compare to the space-grown structure (Figure 2b). The values of radius of gyration for the earth-grown structure fluctuate between 19.9 Å and 21 Å throughout the simulation, while those of the space-grown structure gradually decreased from 20.5 Å to 19.8 Å. Radius of gyration refers to the distribution of the components of an object around an axis, and determines the protein structure compactness, and it is also one way to indicate protein unfolding and denaturation. Galzitskaya and Garbuzynskiy [16] proposed that proteins with the highest value of radius of gyration can be considered to have less tight packing. The results showed that space-grown crystal structure of T1 lipase is less flexible than the earth-grown crystal structure.

To better understand structural variations and conformational flexibility in both proteins, the RMSFs of Cα on the protein backbone were measured to study the fluctuations of each residue over the simulation time. The enhancement of flexibility can be observed in terms of the average interatomic distances between the atoms. The analysis of RMSF is in agreement with the radius of gyration in which the fluctuation of the earth-grown T1 lipase is greater especially at the N-terminal of the structure (Figure 2c). The increasing atomic mobility in the earth-grown structure was dispersed throughout the protein. The peak regions of the RMSF values of the earth-grown structure encompass mainly the sequence regions at positions Ser2–Asn6 (N-terminal), Lys138–Val142 (helix 5), Arg214–Ser220 (the loop before helix 7), and Arg387–Pro388 (C-terminal). The regions Gly275–Asn280 (the ß8–ß9 loop) showed the peak of RMSF value in the both structures. According to Baweja et al. [17], residue located in the inside region of protein structures display low RMSF values, while, loop regions and residues reside on the surface of proteins exhibit higher RMSF values. Interestingly, the comparison of B-factor of both T1 lipase structure is in agreement with our analyses. Aris et al. [7] reported that earth-grown of T1 lipase crystal structure shows higher B-factor value compared to the space-grown crystal structure, indicating higher flexibility in earth-grown structure.

### 2.3. Analysis of the Secondary Structure

**Figure 3:**
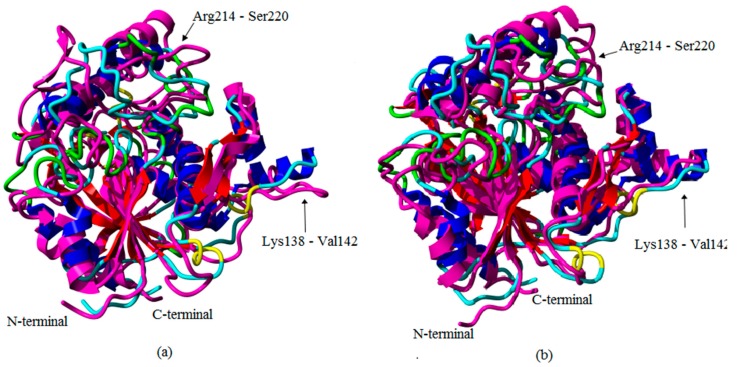
Superimposed T1 lipase structures before and after 20 ns of simulation: (**a**) Earth-grown structure at 0 ns (color in elements) and after 20 ns of simulation (color in magenta); (**b**) Space-grown crystal structure at 0 ns (color in elements) and after 20 ns of simulation (color in magenta). The arrows showed the region with a high flexible value in the earth-grown structure compared with the space-grown structure.

**Figure 4:**
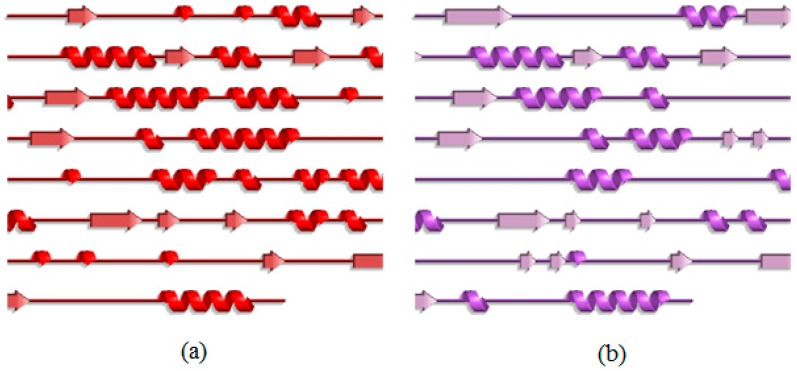
Changes in secondary structure of earth-grown T1 lipase crystal structure before and after 20 ns of simulation: (**a**) Crystal strcuture of earth-grown T1 lipase before simulation; (**b**) Earth-grown T1 lipase after 20 ns of simulation.

**Figure 5:**
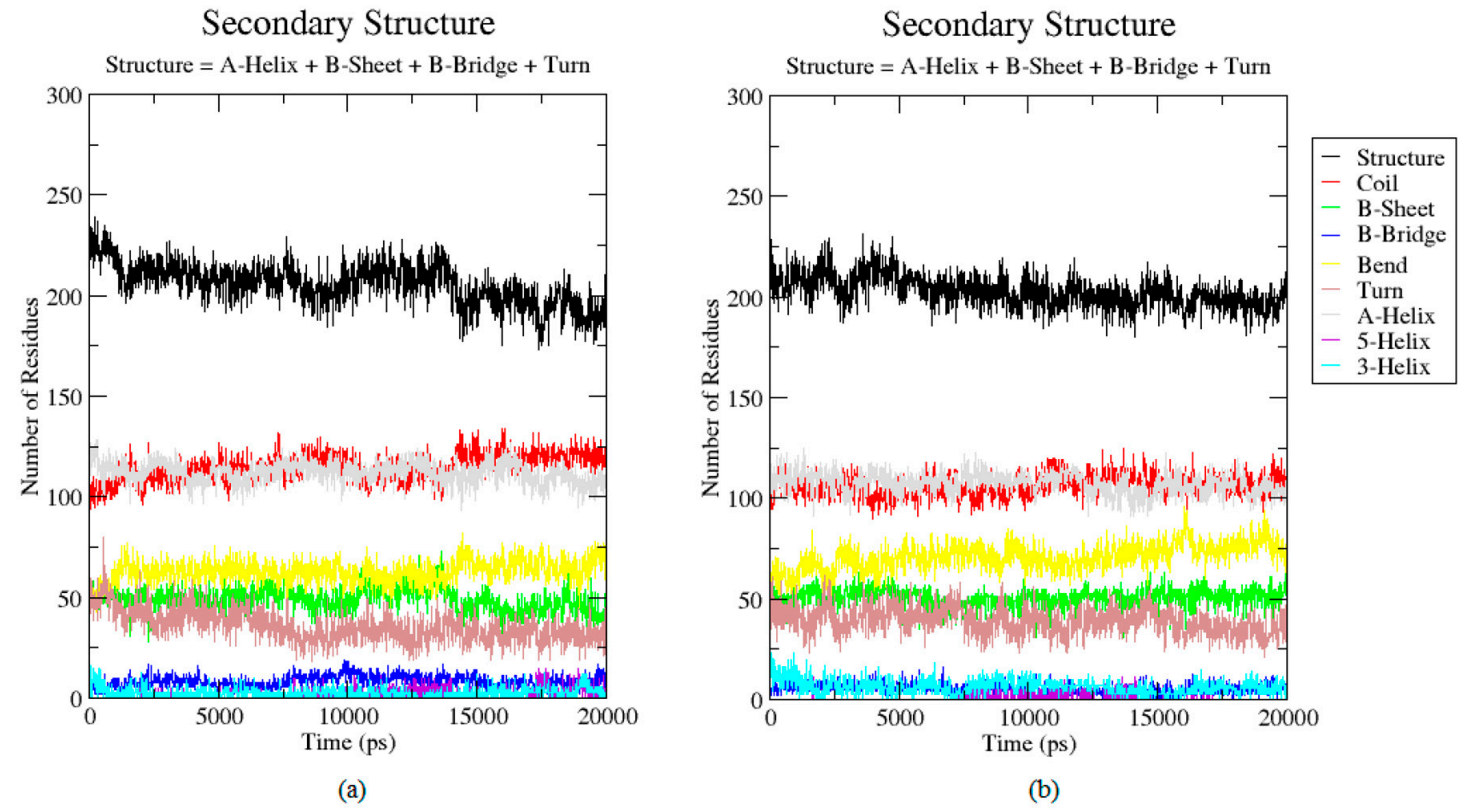
The secondary structure analysis for the earth-grown and space-grown structures: (**a**) Earth-grown structure; (**b**) Space-grown structure.

To describe in more detail of the conformational changes, the secondary structure of both T1 lipase earth-grown and space-grown crystal structures were analyzed. These analyses showed that the flexible region Lys138–Val142 which showed a high RMSF value in the earth-grown structure exhibited structural changes from a stable helix to a random coil during the simulation. The details defined the increasing of the fluctuation in these regions (Figure 3a). As illustrated in Figure 4, the earth-grown structure encountered tremendous changes in secondary structural elements. In Figure 5, detailed information is presented as residue participates in the secondary protein structure. The secondary structures observed during the MD simulation were showed by do dssp module of GROMACS, suggesting conformational changes in both structures in their secondary structural elements. The first general comparison of both analyzed structures confirmed the fluctuation in the secondary structure of both the earth-grown and space-grown structure. In addition, it was observed that the total counts in terms of total α-helix, ß-sheet, ß-bridge, and turn decreased.

### 2.4. Hydrogen Bond and Ionic Interaction Formation and Deformation

**Figure 6:**
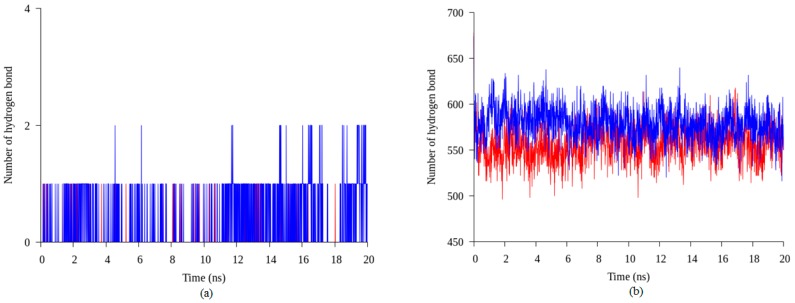
Hydrogen bond interaction in the earth-grown and space-grown T1 lipase structures: (**a**) Hydrogen bond numbers between amino acid Thr306 and Asn304 in the space-grown (blue) and earth-grown (red) structures during 20 ns of simulation; (**b**) Number of hydrogen bonds in the space-grown (blue) and earth-grown (red) structures during 20 ns of simulation.

Hydrogen bonds are important in protein folding, protein structure, and molecular recognition. These bonds are vital in the formation of protein secondary structure namely alpha helices and beta strands, which are the key of protein function. Initial study on hydrogen bond number, in both structures, showed that the space-grown structure of T1 lipase consisted of more hydrogen bonds compared to the earth-grown structure (Table S1). The results of different locations of hydrogen bonds for both crystal structures are shown in Table S2. Based on the initial result of crystal structure, five hydrogen bonds which are found in space-grown structure and absent in earth-grown structure were analyzed during 20 ns of simulation to evaluate the stability of hydrogen bonds formed in both structures. The results indicate that hydrogen bonds between amino acid Thr306 and Asn304 were found to form in almost 90% of the simulation time in space-grown structure with average 0.18 of hydrogen bond numbers per timeframe. In contrast, in the earth-grown structure, this hydrogen bond presents as an isolated hydrogen bond throughout the simulation with average only 0.03 hydrogen bond numbers per timeframe (Figure 6a). The same results were found in other sets of hydrogens between amino acid Glu250–Gln254, Gln39–Asp43, and Asn59–Thr118 which showed that the average of hydrogen bond numbers in the space-grown structure are more than the earth-grown structure (Table S3). Figure 6b showed the total number of hydrogen bond interaction in the space-grown structure are more than the earth-grown structure throughout the simulation. The results suggested that the interaction in the space-grown structure remains intact longer than the interaction formed in the earth-grown structure. Vogt et al. [18] stated that the number of hydrogen bonds and the polar surface are related to the protein thermostability. The fractional polar surface would increase the density of the hydrogen bond with the surrounding water molecules. Myers et al. [19] concluded that the hydrogen bond is crucial in globular protein to stabilize its structure. Efimov and Brazhnikov [20] indicated that the possibility of the hydrogen bond formation could be increased if the solvent accessibility of side chain donors and acceptors are lower. They also suggested that intramolecular hydrogen bonding is favorable for buried residues and becomes less favorable for solvent exposed polar atoms due to the lack of competition of hydrogen bonding with water molecules [20]. However, research by Szilagyi and Zavodsky [21] revealed that the numbers of hydrogen bond were not significantly different in both thermophilic and mesophilic proteins which showed that the numbers of hydrogen bond do not affect the thermostability of protein. Kar and Scheiner [22] classified the hydrogen bond as weak and strong depending on its donor and acceptor which would give a different energy in protein stability. The effect of different types of hydrogen bonds are classified into backbone-backbone, backbone-sidechain, side chain-backbone, and side chain-side chain related to the thermostability of the protein which showed a different free energy production by each component [23].

**Figure 7:**
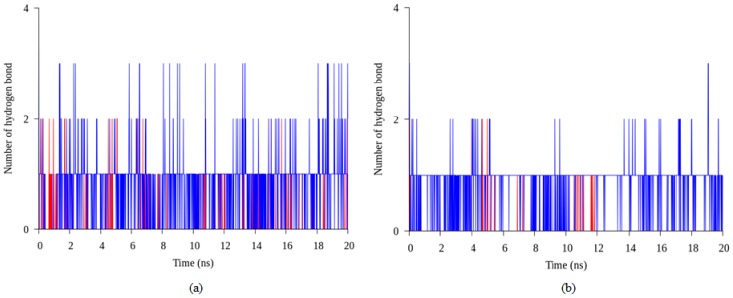
Interaction in earth-grown and space-grown T1 lipase structures: (**a**) Number of interaction between residue Arg230 and Glu226 in the space-grown (blue) and earth-grown (red) structures during 20 ns of simulation; (**b**) Number of interaction between residue Lys229 and Asp178 in the space-grown (blue) and earth-grown (red) structures during 20 ns of simulation.

**Figure 8:**
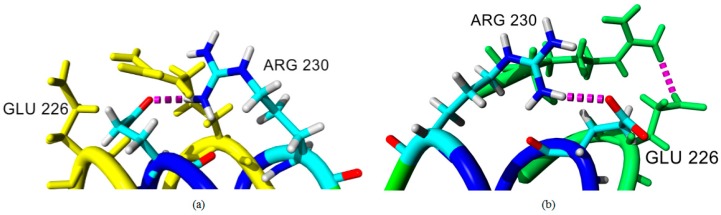
Ion pair interaction in earth-grown and space-grown T1 lipase structures: (**a**) Superpose of earth-grown T1 lipase crystal structure (color in elements) and structure at 20 ns (color in yellow); (**b**) Superpose of space-grown T1 lipase crystal structure (color in elements) and structure after 20 ns of trajectory (color in green). Ion pair interaction is indicated using dotted lines in magenta.

A salt bridge or ionic interaction plays an important role in protein thermostability. The number of ion pairs established in a thermophilic protein are higher than the number of ion pairs in a mesophilic protein indicated that the increasing number of ion pairs correlated relatively in thermostability of the protein [21]. In this study, we observed that the number of ion pair interactions in the earth-grown crystal structure of T1 lipase are relatively higher compared to the space-grown crystal structure. The ionic interaction in chain A and chain B of the earth structure consist of 25 and 22 ion pairs, respectively. While the space-grown structure consists of 22 ion pairs in both chains. However, further analyses, via molecular dynamic simulation, showed that some of the interactions were not stable in the earth-grown structure. On the contrary, the interaction of these amino acids become stronger in the space-grown structure as exemplified in Figure 7a,b, which shows the overall interaction between Arg230–Glu226 and Lys229–Asp178. These four residues were also found to form the largest ion pair networks in both T1 lipase structure. As depicted in Figure 8a,b, interaction between residue Arg230 and Glu226 was found in both crystal structures. However, the interaction in the earth-grown structure was splintered after the simulation. Kumar and Nussinov [24] indicated that the geometry and location of ion interaction in a protein may affect the stability of the protein. The stability of the protein can be dependent on the networked ion pairs, number of ion pairs, and electrostatic interactions. The location of the ion pair on the surface of the protein could be the main element that contributes to the strength of the ion pair [25]. In hyperthermophilic rubredoxin (PFRD-XC4) structure, the surface ion pair between side chains of Lys6 and Glu49 does not contribute to the overall stability of its structure. However, the presence of ion pairs between the amide at the N-terminal and Glu14 was found to have stabilized the structure of *Pyrococcus furiosus* rubredoxin (PFRD-XC4) by 1.5 kcal/mol [26]. Rahman et al. [27] suggested that the construction of new ion pair interactions between the subunit of the glutamate dehydrogenase (GDH) enhanced the thermostability of the protein.

In our study, the distance of ion pair interactions was found to range from 1.67 Å to 2.40 Å and classified as strong salt bridges. Szilagyi and Zavodsky [21] used a distance limits of 4.0 Å for the strongest ion pair connection in their study. The other study, by Kumar and Nussinov [28], demonstrated that most of the ion pairs with distances less than 5 Å are possibly able to stabilize the structure of protein. The number of ion pairs associated with the reduction of the hydrophobic surface could enhance the stability of proteins from hyperthermophiles [29]. In both structures, Arg residues are more likely to participate in ion pair interaction. Pack and Yoo [30] indicated that Arg has higher probability to establish ion pair interaction. Participation of Arg in ion pair interaction could provide more stabilizing effect on exposed part of protein structure. The largest ion pair network was found in both space and earth structures which composed by the residues Lys229, Asp178, Glu226, and Arg230.

Different growth conditions for the crystal structure of T1 lipase presented different information about structural properties, leading to varying hydrogen bonds and ion interactions. Wu et al. [31] indicated that MD simulation revealed that a newly formed hydrogen bond contributing to a reduction in the flexibility of the global structure of proteins. The T1 lipase space-grown structure consisted of a higher number of hydrogen bonds as compared to the earth-grown, hence, it might be the reason why the space-grown crystal structure is less flexible and more stable since it contributes to the protein stability. An additional hydrogen bond, introduced into the lipase *Stenotrophomonas maltophilia,* increased its thermostability. This indicates that hydrogen bond strategy is a powerful approach for improving enzyme stability [31]. Hence, three-dimensional structures of protein play an important role in the manipulation of this strategy in the development of the protein and drug design. This demonstrates the importance of good quality crystals with high resolution, which can provide explicit information and translation of protein structure. Thus, high accuracy with more detailed information obtained from the space-grown crystal structure of T1 lipase provided more accurate structural data for further analysis such as production of a high quality protein with improved characteristics.

The results showed that the space-grown structure have more ordered water formed with more hydrogen bonds than the ground structure. Habash et al. [32] concluded that the interaction of surfaces residues which form crystal lattice interaction lead to the improvement of crystal growth at the molecular level. The reduction of convection in microgravity environments possibly produced better crystals with better arrangements. The reduction of sedimentation rate and convection in microgravity condition may influence the moving of the solution. In other words, low gravity allowed the molecules to be transported by slower diffusion hence allowed more interaction with water and surrounding of the protein [33]. Bogon et al. [34] reported that microgravity suppressed the flowing of fluid and crystal movement which triggered the formation of big crystal of α-crustacyanin. The observation of the growing process of thaumatin crystal under microgravity indicates that their nucleation was more synchronous as compared with crystal grown under earth condition. The microgravity crystal of thaumatin also reported to have better optical properties and resolution with lower mosaic spread. This experiment proved that crystallization under microgravity condition enhanced the properties of protein crystal. On earth, convection could induce rapid mixing of the content in the solution during the crystallization process, meanwhile, the absence of convection encourages gradual diffusion of the salt in the protein solution which triggers nucleation when the critical supersaturation is achieved [35]. In microgravity, the incorporation of molecules into the crystal depends highly on diffusion. The molecules may be allocated in order and the incorporation of impurity may be suppressed. Consequently, the nature of this microgravity environment brings growth to the highly ordered crystals.

## 3. Materials and Methods

### 3.1. Preliminary in Silico Study of Space- and Earth-Grownspace- and Earth-Grown Crystal Structures of T1 Lipase

The thermoalkaphilic T1 lipase of *Geobacillus zalihae* was crystallized under microgravity conditions in collaboration with JAXA under the JAXA-UPM Protein Crystal Growth (PCG) #2 Flight Program. T1 lipases were successfully crystallized under microgravity and earth environment with modified capillary counter diffusion method using reservoir solution composed of 1 M NaCl, 0.1 M NaH_2_PO_4_, 0.1 M KH_2_PO_4_, and 0.1 M MES at pH 6.5. The crystallization temperature was set to 20 °C for both space and earth conditions [7]. In silico molecular modeling was used to identify any differences between the crystal structures of T1 lipase obtained from space and earth conditions. The PDB files of both structures were superimposed using YASARA (Yet another Scientific Artificial Reality Application) software version 10.2.1 [36]. The RMSD and matched atoms were observed and determined. The variations on hydrogen bonds and ion pair interactions formed by the space- and earth-grown structures of T1 lipase were investigated. Hydrogen bonds between donors (H) and acceptors (N and O) were observed with a distance cutoff of 3.5 Å. The existence of ion pair interactions in both the space- and earth-grown crystal structures were observed between negatively charged amino acid (Asp and Glu) and positively charged amino acids (Arg, Lys, and His). Ion interactions formed when carbonyl oxygen atoms of the negatively charged Asp or Glu side chain were found to be within a 4.0 Å distance from nitrogen atoms of positively charged Arg, Lys, and His side chains.

### 3.2. Molecular Dynamic Simulation

Molecular dynamic simulations of recombinant T1 lipase derived from the earth-grown and space-grown crystal structures were performed using the Gromoss96 53a6 force field by using GROMACS version 5.1.2 [37,38]. Before performing the production runs, the systems were solvated in cubic water boxes with spc216 water molecules and sodium ions were added to neutralize the systems. Energy minimization was performed using the steepest descent method to diminish steric clash and unfitting geometry. Prior to simulation, two phases of equilibration were applied to the systems for 100 picosecond (ps) each. The first phase of equilibration was conducted under an NVT (constant Number of particles, Volume, and Temperature) ensemble to stabilize the temperature followed by the NPT ensemble (Number of particles, Pressure, and Temperature) ensemble to stabilize the pressure and density. The productions for both structures were performed at a temperature of 343.15 K (70 °C) for 20 nanoseconds (ns). Both chain A and chain B of the space-grown and earth-grown structures were simulated individually and the average of the structures was determined.

### 3.3. Analysis of MD Simulation Trajectories

Analyses of protein structures such as root mean square deviation (RMSD), root mean square fluctuation (RMSF), radius of gyration, hydrogen bonds, and ion pair interactions were performed using GROMACS simulation package. The RMSD value is the measure of the average distance between the atoms which also indicates structural changes during the simulation. RMSD should be able to differentiate between poor and good quality of structure. A high value of RMSD can be concluded to exhibit poor quality of protein structure [3]. RMSD calculations were performed using the starting structure of each simulation as a reference. For hydrogen bond calculation, a donor-acceptor cutoff distance of 3.5 Å and acceptor-donor-hydrogen bond angle cutoff of 30° were examined. Secondary structure analysis was performed using dssp program. Visual analysis of structures and preparation of figures was carried out using yasara, xmgrace and gnuplot.

## 4. Conclusions

The crystal structures of T1 lipase derived from the same crystallization method under space and earth condition showed dissimilarity in both hydrogen bond numbers and ion pair interactions. Comparative analysis of the two structures revealed that T1 lipase crystal structure crystallized under a space environment exhibited a higher number of hydrogen bonds than the earth-grown structure. Correspondingly, the space-grown crystal offers clearer structural information. Higher accuracy of structural information is crucial in the development of structure-based design and redesign of high quality proteins with certain characteristics to fit in industrial applications. Further analysis by MD simulations concluded that earth-grown T1 lipase structure displayed higher flexibility than the space structure. A molecular dynamic simulation allowed for a detailed comparison of the structures and confirmed the differences that cannot be demonstrated in a wet lab.

## Supplementary Materials

The following are available online. Figure S1: Superimposed of space-grown (blue) and earth-grown (red) T1 lipase crystal structures. Figure S2: Superimposed of space-grown (blue) and earth-grown (red) of T1 lipase structure showed a region with different conformation at residue Asp175. Table S1: Total number of hydrogen bond of T1 lipase space- and earth-grown crystal structures. Table S2: Different location of hydrogen bond in T1 lipase space-grown crystal compared to earth-grown crystal structures. Table S3: Average number of hydrogen bond between given residues in earth-grown and space-grown structures throughout 20 ns of simulation.
