# Supplementary material for: Molecular Dynamic Simulation of Space and Earth-Grown Crystal Structures of Thermostable T1 Lipase Geobacillus zalihae Revealed a Better Structure
Source: Molecules. 2017 Sep 25;22(10):1574. doi: 10.3390/molecules22101574 (PMC6151610; doi:10.3390/molecules22101574)
Supplement: Supplementary file 1 [file molecules-22-01574-s001.pdf]

Molecular Dynamic Simulation of space-grown and earth-grown crystal structures of thermostable T1 lipase *Geobacillus zalihae* revealed a better structure.

Siti Nor Hasmah Ishak[1], Sayangku Nor Ariati Mohamad Aris[1,3],Khairul Bariyyah Abd Halim[7], Mohd Shukuri Mohamad Ali[1,4], Thean Chor Leow [1,3,5], Nor Hafizah Ahmad Kamarudin [1,3], Malihe Masomian[1,2] and Raja Noor Zaliha Raja Abd. Rahman[1,2,5,6,*]

1 Enzyme and Microbial Technology Research Centre, Faculty of Biotechnology and Biomolecular Sciences, Universiti Putra Malaysia, 43400 UPM Serdang, Selangor, Malaysia.
2 Department of Microbiology, Faculty of Biotechnology and Biomolecular Sciences, Universiti Putra Malaysia, 43400 UPM Serdang, Selangor, Malaysia.
3 Department of Cell and Molecular Biology, Faculty of Biotechnology and Biomolecular Sciences, Universiti Putra Malaysia, 43400 UPM Serdang, Selangor, Malaysia.
4 Department of Biochemistry, Faculty of Biotechnology and Biomolecular Sciences, Universiti Putra Malaysia, 43400 UPM Serdang, Selangor, Malaysia.
5 Institute of Bioscience, Universiti Putra Malaysia, 43400 UPM Serdang, Selangor, Malaysia.
6 Laboratory of Halal Science Research, Halal Products Research Institute, Universiti Putra Malaysia, 43400 UPM Serdang, Selangor, Malaysia
7 Kuliyyah of Science, International Islamic University Malaysia, P.O. Box 10, 50728 Kuala Lumpur, Malaysia
* Correspondence: rnzaliha@upm.edu.my; Tel.: +603-8946-7592

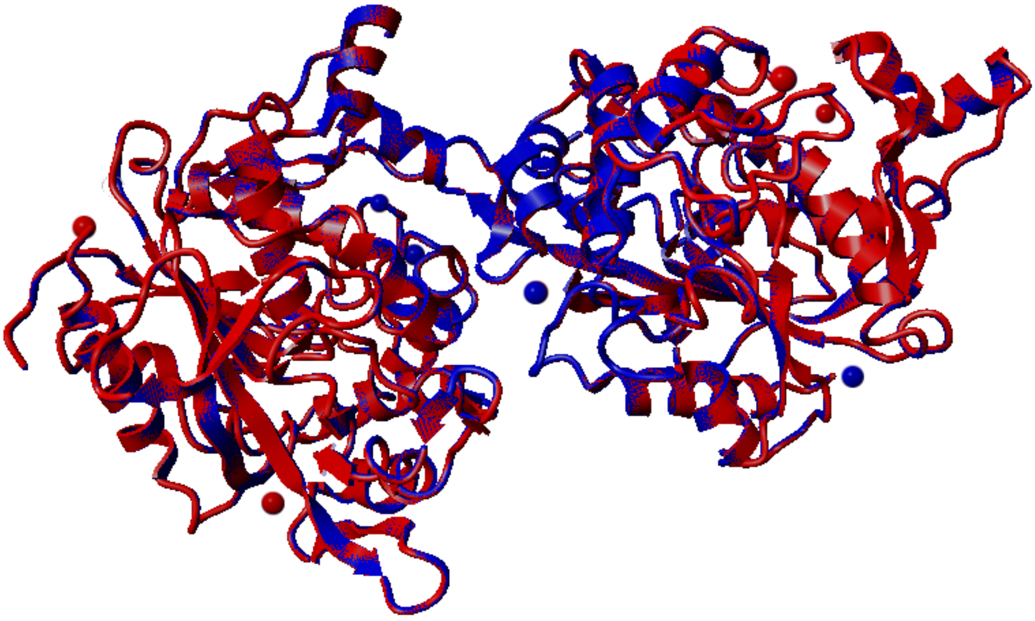

Figure S1. Superimposed of space-grown (blue) and ground-grown (red) T1 lipase crystal structures.

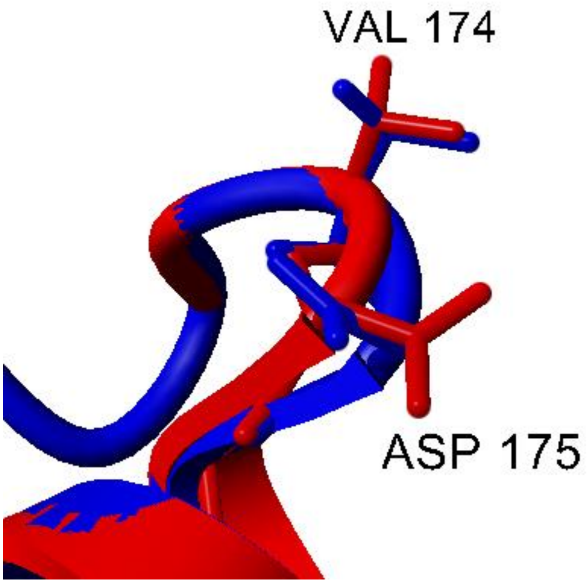

Figure S2. Superimposed of space-grown (blue) and ground-grown (red) of T1 lipase structure showed a region with different conformation at residue Asp175.

Table S1. Total number of hydrogen bond of T1 lipase space-grown and earth-grown crystal structures.

	**Number of hydrogen bonds better than 6025 kJ/mol**
Space-grown	1834
Earth-grown	1800

Table S2. Different location of hydrogen bond in T1 lipase space-grown crystal compared to earth-grown crystal structures.

**Earth-grown structure**	**Space-grown structure**
O Leu 208 < N Trp 211	ND2 Asn 42> O Thr 48
N Asn 321 > OD1 Asn 321	O Glu 250 < N Gln 254
OD1 Asn 367 < N Ser 369	O Thr 342 < NZ Lys 344
	OD1 Asn 304 < OG1 Thr 306
	NH1 Arg 34 > O Phe 370
	NE2 39 Gln > OD1 Asp 43
	OD1 Asn 59 < OG1 Thr 118
	O Glu 226 < NH1 Arg 230

Hydrogen bond which are same in both structures are not shown. The symbols of > represent 'donated to' while < represent 'accepted from'.

Table S3. Average number of hydrogen bond between given residues in ground-grown and space-grown structures throughout 20ns of simulation.

Residues	Ground-grown structure	Space-grown structure
Glu250 and Gln254	0.16	0.60
Asn304 and Thr306	0.03	0.18
Gln39 and Asp43	0.59	0.88
Asn59 and Thr118	0.43	0.66
